# Distinct actin cytoskeleton behaviour in primary and immortalised T-cells

**DOI:** 10.1242/jcs.232322

**Published:** 2019-09-04

**Authors:** Huw Colin-York, Sudha Kumari, Liliana Barbieri, Lena Cords, Marco Fritzsche

**Affiliations:** 1 MRC Human Immunology Unit, Weatherall Institute of Molecular Medicine, University of Oxford, Headley Way, Oxford OX3 9DS, UK; 2 Koch Institute of Integrative Cancer Research, Massachusetts Institute of Technology, Cambridge 02139, USA; 3 Kennedy Institute for Rheumatology, University of Oxford, Roosevelt Drive, Oxford OX3 7LF, UK

**Keywords:** Actin cytoskeleton, Jurkat cells, Immune synapse, Primary T-cells

## Abstract

Cytoskeletal actin dynamics are crucial for the activation of T-cells. Immortalised Jurkat T-cells have been the model system of choice to examine and correlate the dynamics of the actin cytoskeleton and the immunological synapse leading to T-cell activation. However, it has remained unclear whether immortalised cellular systems, such as Jurkat T-cells can recapitulate the cytoskeletal behaviour of primary T-cells. Studies delineating the cytoskeletal behaviour of Jurkat T-cells in comparison to primary T-cells are lacking. Here, we employ live-cell super-resolution microscopy to investigate the cytoskeletal actin organisation and dynamics of living primary and immortalised Jurkat T-cells at the appropriate spatiotemporal resolution. Under comparable activation conditions, we found differences in the architectural organisation and dynamics of Jurkat and primary mouse and human T-cells. Although the three main actin network architectures in Jurkat T-cells were reminiscent of primary T-cells, there were differences in the organisation and molecular mechanisms underlying these networks. Our results highlight mechanistic distinctions in the T-cell model system most utilised to study cytoskeletal actin dynamics.

## Abstract (precis)

**Summary:** The emerging idea that the cytoskeletal and biophysical principles are preserved in primary cells and transformed cell lines, and the two can be used to interchangeably examine synaptic actin characteristics, needs careful reconsideration.

## Introduction

A healthy actin cytoskeleton is crucial for T-cell function. The actin machinery integrates T-cell receptor (TCR) signalling and biophysical mechanisms to coordinate the activation of T-cells at the immunological synapse (IS), for T-cell activation, function and differentiation (Colin-York et al., 2019a; Fritzsche et al., 2017; Roy and Burkhardt, 2018). Actin dysregulation results in aberrant IS organisation and immune T-cell dysfunction (Beemiller et al., 2012; Ritter et al., 2015). Immortalised cell systems have been the model of choice to examine actin behaviour at the IS (Carisey et al., 2018; Colin-York et al., 2019b; Comrie et al., 2015; Kaizuka et al., 2007; Rak et al., 2011), owing to their easy transducibility with fluorescent or functional reporter constructs and relatively large size optimal for microscopic visualisation. However, to what extent these cells recapitulate the cytoskeletal behaviour of primary cells remains unclear.

Here, we found differences in the actin organisation and dynamics between Jurkat cells, an extensively utilised immortalised T-cell system, and primary mouse and human T-cells at comparable activation conditions. Consequently, the emerging idea that the cytoskeletal and biophysical principles are preserved in primary and transformed cell lines, and that the two can be used to interchangeably examine synaptic actin characteristics, needs careful reconsideration.

## RESULTS AND DISCUSSION

**Fig. 1.**
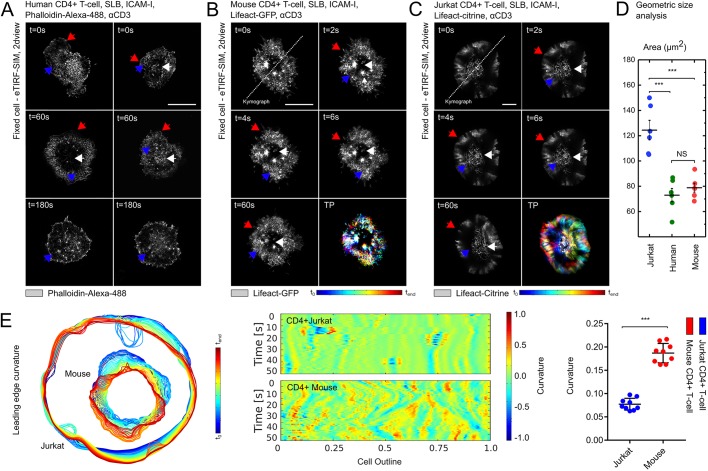
**Distinct actin cytoskeleton architecture in primary and immortalised T-cells.** (A–C) Representative TIRF-SIM images of fixed human CD4^+^ T-cells fluorescently labelled with phalloidin-Alexa-488 (A), live mouse CD4^+^ T-cells expressing F-actin (Lifeact-GFP; B), and Jurkat CD4^+^ T-cells expressing Lifeact-citrine at the basal membrane (C) showing the dynamics within 3 min after contact with the activating SLB. The three characteristic F-actin architectures lamellipodium (red arrows), lamellum (blue arrows) and ramified actin network (white arrow) are visible in the three T-cell types. (D) Geometric size analysis of the contact interface in human, mouse and Jurkat CD4^+^ T-cells in response to the activating SLB. Quantitative differences were observed in the geometric size analysis when comparing Jurkat CD4^+^ T-cells (blue) with primary human CD4^+^ (green) and mouse CD4^+^ T-cells (red) (***P<0.0001) but not between primary human CD4^+^ and mouse CD4^+^ T-cells (NS, *P*>0.9). (E) Analysis of the lamellipodial leading edge curvature for both primary mouse CD4^+^ and Jurkat CD4^+^ T-cells after contact with the activating SLB. Quantitative differences were observed when comparing Jurkat CD4^+^ T-cells (blue) with primary mouse CD4^+^ T-cells (red); ***P<0.0001. Further details are provided in the text. All scale bars: 5 µm.

We employed high-speed live-cell super-resolution microscopy in combination with a supported lipid bilayer (SLB) system to compare the actin organisation and dynamics during early phases of T-cell activation. Quantitative comparison of calcium (Ca^2+^) triggering of large T-cell ensembles of all three cellular systems did not indicate significant statistical differences in the Ca^2+^-triggering fractions but a slowdown in the Ca^2+^ response time of Jurkat CD4^+^ T-cells compared to primary CD4^+^ T-cells (Fig. S1). Under the same experimental conditions, high-resolution optical total internal reflection fluorescence (TIRF) and structured illumination microscopy (SIM) showed apparent differences in the morphology of the actin network at the IS (Fig. 1A–C and Movies 1 and 2). Although the three previously reported actin architectures, including the lamellipodium, the lamellum and the ramified actin network, were present in all three cell systems to different degrees (Table 1) (Fritzsche et al., 2017), only Jurkat T-cells displayed occasional actin arcs (Murugesan et al., 2016) (data not shown) and larger IS contact areas, perhaps due to their overall larger size (Fig. 1D). The lamellar leading edge was more dynamic in mouse primary T-cells, as reflected by significantly higher mean curvature magnitude and persistent fluctuations compared to those in the Jurkat T-cells (Fig. 1E). These data indicated that the cortical actin dynamics are different between primary T-cells and Jurkat T-cells.

**Table 1.**
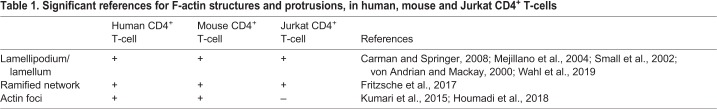
**Significant references for F-actin structures and protrusions, in human, mouse and Jurkat CD4^+^ T-cells**

**Fig. 2.**
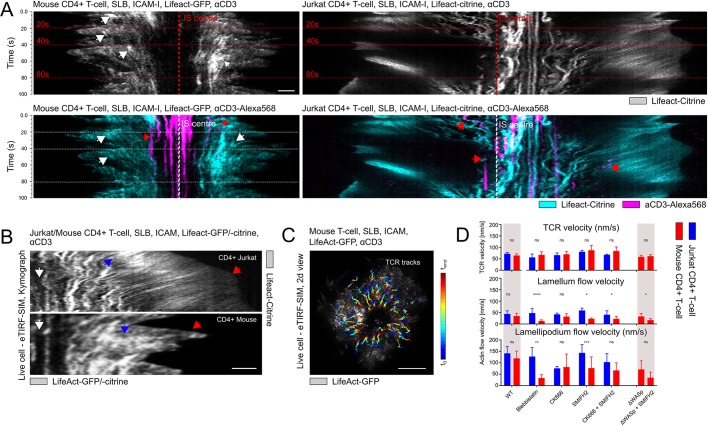
**Distinct actin cytoskeleton dynamics in primary and immortalised T-cells.** (A) Kymographs comparing F-actin-expressing live mouse (Lifeact-GFP, white arrows) and Jurkat CD4^+^ T-cells (Lifeact-citrine) at the basal membrane within 3 min after contact with the activating SLB with and without fluorescent label of anti-CD3-Alexa-568 (red arrow). (B) Kymographs comparing live mouse and Jurkat CD4^+^ T-cells expressing F-actin show immobile F-actin features (white arrows), dynamic F-actin (blue arrows), and distinct leading edges in live mouse and Jurkat CD4^+^ T-cells (red arrows). (C) Representative tracking of the TCR over time in live mouse CD4^+^ T-cells expressing F-actin (Lifeact-GFP) with early-to-late time-points scaled in cold-to-warm colours. (D) Quantification of actin and TCR flow velocities for multiple experimental conditions. No quantitative differences were observed in the TCR flow velocities (ns, *P*>0.9) across experimental conditions as well as comparing between primary mouse CD4^+^ and Jurkat CD4^+^ T-cells (ns, *P*>0.9). In contrast, quantitative differences were observed in actin flow velocities (**P<0.001) under experimental conditions as well as when comparing primary mouse CD4^+^ and Jurkat CD4^+^ T-cells (**P<0.001). Further details are provided in the text. Scale bars: 1 μm (A,B), 5 μm (C).

**Fig. 3.**
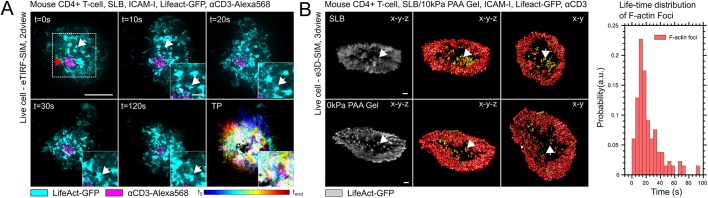
**Distinct actin cytoskeleton protrusions in primary T-cells.** (A) Mouse CD4^+^ T-cells form actin foci during activation (white arrows) in the periphery of the IS (red arrows). (B) Actin foci (white arrows and yellow in 3D reconstruction in images) form in the centre of the contact interface with a well-defined life-time distribution (bar graph). Scale bars: 5 µm (A), 1 µm (B).

To examine actin dynamics, we next imaged the synaptic actin network of the two different T-cell systems: primary mouse CD4^+^ T-cells and Jurkat CD4^+^ T-cells (Fig. S2). Consistent with the curvature quantifications, we found that the cortical network in primary cells underwent undulations with an average frequency of 0.1 Hz, while it was stable in Jurkat CD4^+^ T-cells (Fig. 2A). This distinction led us to further characterise the molecular differences underlying the cortical actin network (Fig. 2B) and their implications on TCR microcluster motility at the synapse (Fig. 2C and Movies 3 and 4). When comparing primary mouse CD4^+^ with Jurkat CD4^+^ T-cells, we did not detect any significant changes in the TCR flow velocity across the lamellipodium and lamellum in response to pharmacological treatments targeting the main actin nucleation pathways, i.e. inhibition of the Arp2/3 complex or of formins in response to treatment with CK-666 or SMIFH2, respectively (Fig. 2D). To this end, we quantified the retrograde filamentous actin (F-actin) flow in response to these pharmacological treatments (Fig. 2D and Figs S3 and S4). The CK-666 and SMIFH2 treatments affected the integrity and F-actin flow velocities of the lamellipodium and lamellum, and of ramified architectures in primary mouse CD4^+^ and Jurkat CD4^+^ T-cells. Particularly strong changes were observed in response to the combination of the inhibition of both Arp2/3 complex and formins. We also blocked the myosin-II motor activity via photo-resistant Blebbistatin because myosin activity also regulates F-actin flow (Comrie et al., 2015). While the velocity was comparable between the primary and Jurkat T-cells under control conditions, we found that the F-actin flow was mainly generated by myosin-II in primary T-cells and by actin nucleation and/or polymerisation in the Jurkat T-cells. Furthermore, the F-actin dynamics were dominantly localised to lamella in the case of primary T-cells but to lamellipodia in the Jurkat T-cells (Fig. 2B) (Comrie et al., 2015; Murugesan et al., 2016). In addition, the F-actin features, termed ‘actin foci’ (Kumari et al., 2015), were visible in primary (Fig. 3A) but not in Jurkat T-cells (Figs S2 and S3), albeit they did not influence the F-actin flow velocity. Actin foci have previously been reported to localise with TCR microclusters (Kumari et al., 2015) and their life-time was comparable with F-actin turnover at the synapse (Fig. 3B) (Fritzsche et al., 2017). Interestingly, the lack of foci in WASP-deficient primary T-cells did not affect the velocity of TCR microcluster motility at the synapse (Fig. 2D). Notably, the network structure and integrity of the actin architectures at the synapse were affected by the different pharmacological treatments (Figs S2 and S3), whereas TCR microcluster velocity was independent of myosin-II and/or actin polymerisation in either of the cellular systems, indicating that further studies are warranted to investigate the molecular mechanism of TCR microcluster motility at the synapse.

Together, these data highlight that primary T-cells and Jurkat T-cells, indeed, have key differences in their actin architectural and biophysical makeup. Primary and immortalised Jurkat CD4^+^ T-cell lines exhibited, to varying degrees, the three previously reported actin architectures (Table 1) but actin foci were only present in primary T-cells. Analysis of the actin network dynamics revealed a stronger dependence on myosin-II in the case of primary T-cells, and on actin turnover dynamics in the case of Jurkat T-cells. Both T-cell types displayed robust TCR motility at the synapse when the two main actin nucleators, formins or Arp2/3-complex, or the microfilament motor myosin-II were inhibited.

Historically, Jurkat T-cell lines have been used as a substitute for primary T-cells for cytoskeleton imaging due to the ease of their manipulation, but our data highlight that these cells have an actin organisation and dynamics that is distinct from the primary T-cells. Given that actin dynamics is crucial in mediating the early steps of T-cell activation at the synapse (Roy and Burkhardt, 2018), care should be taken when extrapolating results obtained when using either of the two cell systems. It is noteworthy that Jurkat T-cells have also served as a valuable model system to investigate early TCR dynamics and signalling (Abraham and Weiss, 2004), and the TCR signalling pathways appear to be comparable between primary and Jurkat T-cells. These similarities might be due to the robustness of TCR dynamics in the two cells types (Fig. 2D), or might emanate from a lack of negative regulators of TCR signalling, such as SHIP-1 and PTEN, in Jurkat T-cells (Abraham and Weiss, 2004), thereby compensating for the architectural deficits in the actin cytoskeleton. Mechanisms by which Jurkat T-cells conserve TCR signalling despite differences in their cytoskeletal make-up will be a topic of future research.

## MATERIALS AND METHODS

### Cell culture

Primary human and mouse CD4^+^ T-cells, and immortalised Jurkat CD4^+^ T-cells were cultured in sterile RPMI-1640 medium (catalog no. R8758, Sigma-Aldrich) supplemented with 10% FCS, 2 mM L-glutamine (Sigma-Aldrich), 1 mM sodium pyruvate (Sigma-Aldrich), 10 mM HEPES (Sigma-Aldrich), and 1% penicillin/streptomycin-neomycin solution (Sigma-Aldrich). Cells were maintained at 37°C and 5% CO_2_ during culturing and, typically, kept at a density of 5–9×10^5^ cells/ml. Plasmid transfection and lentivirus infections were used to generate cell lines expressing the constructs Lifeact-citrine and Lifeact-GFP.

### Isolation of primary human and mouse CD4^+^ T-cells

Human or murine CD4^+^ T-cells were prepared using the CD4^+^ T Cell Isolation kit (130-096-533 and 130-104-454, respectively) according to the manufacturer's recommendations (Miltenyi Biotec).

### Fixation of primary human CD4^+^ T-cells

Primary CD4^+^ T-cells were washed and resuspended in cytoskeleton buffer (50 mM imidazole, 50 mM KCl, 0.5 mM MgCl_2_, 0.1 mM EDTA, and 1 mM EGTA pH 6.8) at a concentration of 2.5×10^6^ cells/ml. Coverslips were submerged in 1 ml of cytoskeleton buffer in six-well plates, and 100 μl of cells was dropped evenly across the surface of the coverslip; further details are provided in Fritzsche et al. (2017). Cells were incubated at room temperature, after which the cytoskeleton buffer was replaced with 1 ml of cytoskeleton buffer supplemented with 0.25% glutaraldehyde and 0.5% Triton X-100; care was taken not to disturb cells attached to the coverslips. Samples were fixed for 5 min at room temperature before they were washed in 2×1 ml of cytoskeleton buffer and covered with 1 ml of cytoskeleton buffer supplemented with 33 nM Alexa Fluor 488–Phalloidin (Life Technologies) for 30 min. Cells were finally washed three times with 1 ml of cytoskeleton buffer before imaging.

### Stable Jurkat T-cells

Jurkat-derived CD4^+^ T-cells stably expressing LifeAct-citrine were generated by using a lentiviral transduction strategy. HEK-293 cells were plated in six-well plates at 3×10^5^ cells/ml, 2 ml per well in Dulbecco's modified Eagle medium (Sigma-Aldrich) +10% FCS. Cells were incubated for 24 h at 37°C and 5% CO_2_ before transfection with the lentiviral packaging vectors p8.91 and pMD.G (0.5 μg/ well), and the relevant pHR-SIN lentiviral expression vector using GeneJuice (Merck Millipore) as per the manufacturer's instructions. Supernatants were harvested 48 h after transfection and filtered using a 0.45-μm Millex-GP syringe filter unit to remove detached HEK-293 cells. Of this virus-containing medium, 3 ml was added to 1.5×10^6^ Jurkat T-cells in 3 ml of supplemented RPMI medium. After 48 h, cells were moved into 10 ml of supplemented RPMI and passaged as normal.

### Preparation of supported lipid bilayers

Supported lipid bilayers (SLBs) were prepared by spin-coating. A solution of 1 mg/ml total dipalmitoylphosphatidylcholine (DOPC, Avanti Polar Lipids, Alabaster, AL) was spin-coated at 3200 rpm onto a clean 25 mm #1.5 glass coverslip (VWR, UK). The resulting lipid film was hydrated using SLB buffer [150 mM NaCl, 10 mM 4-(2-hydroxyethyl)-1-piperazineethanesulfonic acid (HEPES) pH 7.4] and washed several times. Glass coverslips were cleaned using piranha solution (sulfuric acid:hydrogen peroxide diluted 3:2) for 2 h and stored for <1 week. SLBs were loaded with 10 μg/ml human and mouse anti-CD3ε (OKT3 and 2C11, respectively) for the activation of primary and Jurkat T-cells.

### Live-cell super-resolution extended TIRF-SIM

Extended total internal reflection fluorescence structured illumination microscopy (TIRF-SIM) was performed using 488-nm laser (Coherent, SAPPHIRE 488–500) and combined and passed through an acousto-optic tuneable filter (AOTF, AA Quanta Tech, AOTFnC-400.650-TN). The beam was then expanded and sent into a phase-only modulator, consisting of a polarisation beam splitter, an achromatic half-wave plate (Bolder Vision Optik, BVO AHWP3) and a ferroelectric spatial light modulator (SLM; Forth Dimension Displays, SXGA-3DM). Light diffracted by the grating pattern displayed on SLM passes through a polarisation rotator – composed of a liquid crystal variable retarder (LC, Meadowlark, SWIFT) and an achromatic quarter-wave plate (Bolder Vision Optik, BVO AQWP3) – which can rotate the linear polarisation orientation of the diffracted light for different wavelengths to maintain s-polarization, thereby maximising the pattern contrast for all pattern orientation. The desired light of ±1 diffraction order was isolated from all other high-order diffraction light by a hollow barrel mask driven by a galvanometer optical scanner (Model 623OH, Cambridge Technologies, Bedford, MA), and then they were imaged onto the back focal plane of the high-NA objective (Olympus Plan-Apochromat×100 Oil-HI 1.57NA) as two spots at opposite sides of the pupil. After collimated by the objective, the two beams interfered at the interface between coverslip and sample at an intersection angle that was larger than the critical angle for total internal reflection. The generated evanescent standing wave of excitation intensity penetrated axially ∼100 nm into the sample, and was laterally modulated as a sinusoidal pattern that was a low-pass filtered and demagnified image of the grating pattern displayed on the SLM. The resulting fluorescence was collected by the same objective and separated from excitation light by a dichroic mirror, and finally imaged onto a sCMOS camera (Hamamatsu, Orca Flash 4.0 v2 sCMOS), where the structured fluorescence raw data were recorded. The cell samples were imaged inside a micro-incubator (H301, okolab, Naples, Italy) maintaining the physiological conditions of 37°C and 5% CO_2_.

For each time point, three raw images were acquired at successive phase steps of 0, 1/3 and 2/3 over a period of the sinusoidal illumination pattern. This process was then repeated with the sinusoidal excitation pattern rotated by +120° or −120° with respect to the first orientation. As the excitation pattern was conjugated to the grating image displayed on SLM, the phase stepping and pattern rotation could be accomplished by translating and rotating the grating image accordingly. A total of nine raw images was acquired for a single excitation wavelength before being switched to the next and then this acquisition procedure was repeated for each time point. Finally, the raw images were processed and reconstructed into SIM images. TIRF-SIM data were acquired from at least 50 individual cells over the course of at least three independent experiments.

### Pharmacological treatment

Pharmacological actomyosin-specific reagents, such as the Arp2/3 complex inhibitor CK-666 (Merck Bio-sciences, UK), the formin FH2 domain inhibitor SMIFH2 (Sigma-Aldrich, UK), and the photo-resistant myosin inhibitor Blebbistatin (Milipore) were added to the culture medium at concentrations of 100 μM, 40 μM and 100 μM, respectively. Cells were left to incubate between 30 s and 30 min, as indicated in the corresponding experiment description. Notably, inhibitors were also present at the same concentration in the imaging medium. We could not detect any significant differences in T-cells in the presence of DMSO for the different experimental conditions.

### F-actin flow analysis

Commonly applied kymograph analysis was employed to quantify the velocity of F-actin flow for the different experimental conditions by using open-source software ImageJ.

### T-cell receptor motion analysis

Tracking of T-cell receptor clusters was performed by using custom-written Python code based on the tracking library known as Trackpy. The algorithm first located circular features of a user-defined size and intensity range in each frame of the time-lapse (tp.batch). By defining a minimum displacement between frames and a minimum track length, the code links individual localisations into tracks allowing the TCR velocity to be calculated (tp.link_d.f. and tp.filter_stubs).

### Curvature analysis

Curvature analysis of the outer leading edge of primary mouse CD4^+^ and Jurkat CD4^+^ T-cells was performed using open-source QuimP software for ImageJ.

### Ca^2+^ ensemble analysis

Primary mouse and Jurkat T-cells were labelled with 4 µM Fluo-4 AM (Molecular Probes, Invitrogen). For Fluo-4 AM labelling, cells were incubated with the dye for 30 min at room temperature in RPMI (Sigma-Aldrich) without supplements but containing 2.5 mM probenecid. Cells were then washed three times with HBS and finally resuspended in HBS containing 2.5 mM probenecid before addition to the microscope sample container with the prepared microscope coverslip. Cells were imaged at 37°C and 5% CO_2_ using a 10× air objective on a spinning disk confocal microscope (Zeiss Cell Observer Spinning Disc Confocal), with 488 nm laser excitation and fluorescence detection at ∼530 nm, and with an exposure time of 500 ms and a time between frames of 500 ms for 1000 frames. We used a modified version of CalQuo^2^ (Lee et al., 2017), to detect single-cell landing events on the SLB surfaces, and to record fluorescence intensities over time at the coverslip surface. All cells were individually analysed and the fraction of triggering was determined from the total number of cells detected after landing. For the experimental conditions, Ca^2+^ data were acquired in at least 1000 individual cells over the course of at least three independent experiments.

### Geometric size ensemble analysis

Custom-written MATLAB analysis was employed to quantify the contact area in large ensembles of activating primary mouse and Jurkat T-cells. Following the Ca^2+^ ensemble measurements, we modified the CalQuo^2^ algorithms to compute the surface area (Lee et al., 2017) and its corresponding radius for the autodetected T-cells using the CalQuo detection algorithms and pre-implemented MATLAB functions (Fritzsche et al., 2015). All cells were individually analysed and the described parameters were determined from the total number of cells detected during the formation of the immunological synapse. For the experimental conditions, geometric size data were acquired in >1000 individual cells over the course of at least three independent experiments.

### Statistics

For normally distributed data, the geometric mean and standard deviation (±s.d.) was calculated. Statistical comparison of normally distributed data was carried out using unpaired *t*-test and significance was denoted as *P<0.01, **P<0.001, and ***P<0.0001. Outliers outside the normally distributed data were not considered in the significance tests but are included in all plots. For all experimental conditions and analysis, TIRF-SIM data were acquired in >50 individual cells over the course of at least three independent experiments. Ca^2+^ imaging and geometric size data were acquired using >1000 individual cells over the course of at least three independent experiments.

## Supplementary Material

Supplementary information
