# Supplementary material for: Distinct actin cytoskeleton behaviour in primary and immortalised T-cells
Source: J Cell Sci. 2019 Sep 4;133(5):jcs232322. doi: 10.1242/jcs.232322 (PMC6898998; doi:10.1242/jcs.232322)
Supplement: Supplementary information [file joces-133-232322-s1.pdf]

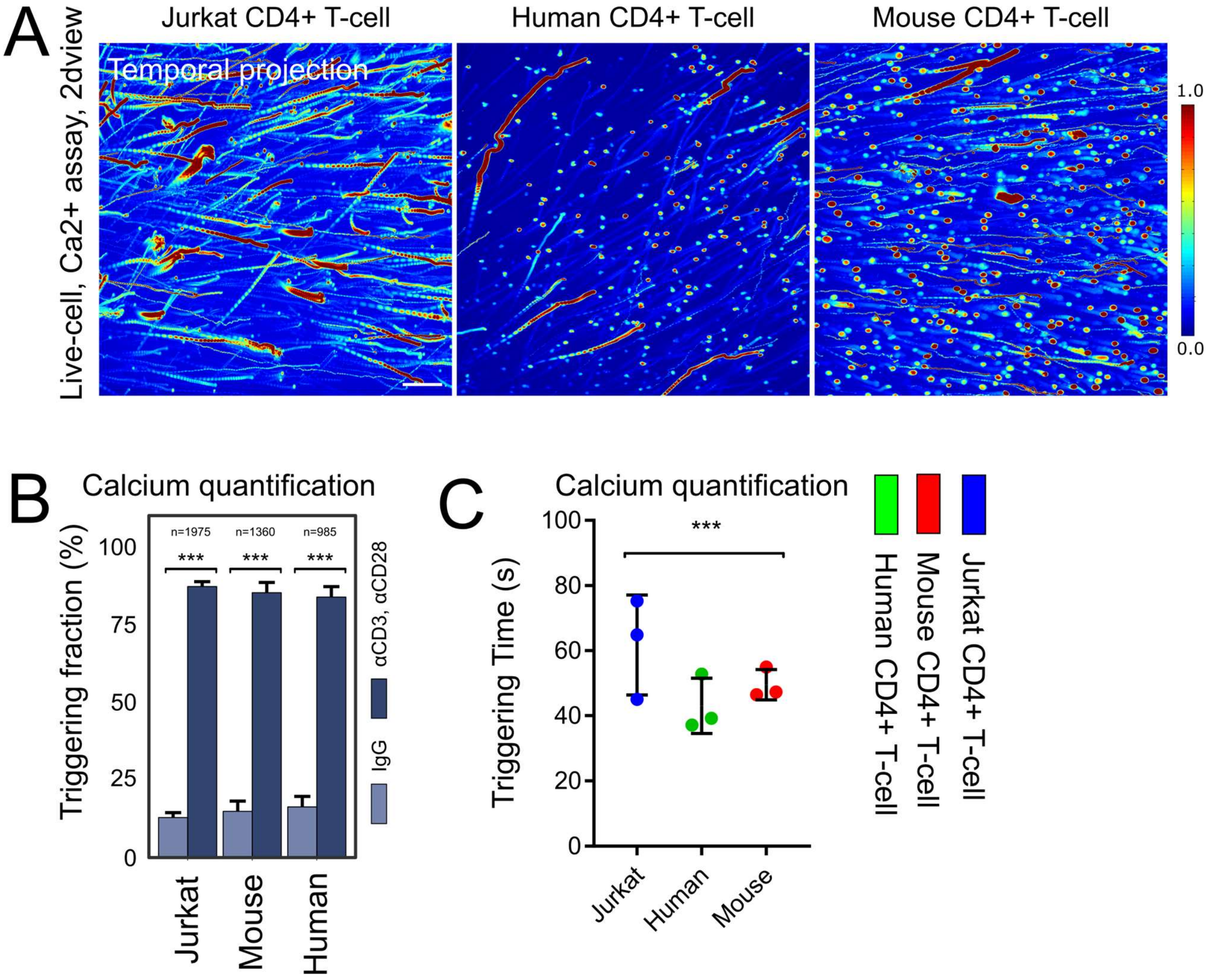

Fig. S1: Calcium response measurements. (**A**) Representative temporal projection of calcium assays computed by superimposing temporal frames of fluorescence intensities of large ensembles of Jurkat CD4+ T-cells, Human CD4+, and Mouse CD4+ T-cells loaded with calcium dye Fluo-4 in response to activating SLBs presenting ICAM-1 and aCD3. Warm to cold colour represent high to low fluorescence intensities. Scale bar is 100 µm. (**B,C**) Quantifications of calcium triggering fractions and triggering times for Jurkat CD4+ T-cells (blue), Human CD4+ (green), and Mouse CD4+ (red) T-cells.

Journal of Cell Science • Supplementary information

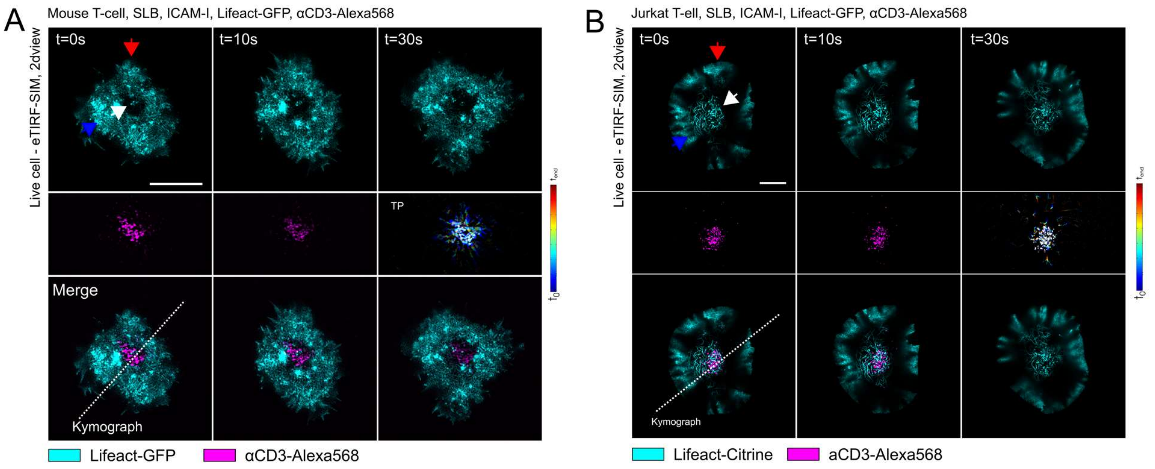

Fig. S2: Distinct actin cytoskeleton behaviour in primary CD4+ Mouse and immortalised CD4+ Jurkat T-cells. Representative TIRF-SIM images of the dynamics of (**A**) live Mouse CD4+ T-cells expressing F-actin (Lifeact-GFP) and (**B**) Jurkat CD4+ T-cells expressing Lifeact-citrine and fluorescently labelled aCD3-Alexa568 at the basal membrane within 3 min after contact with the activating SLB. The three characteristic F-actin networks including the lamellipodium (red arrow), lamellum (blue arrow), and ramified actin network (white arrow) are visible in the Mouse CD4+ and Jurkat CD4+ T-cells. Scale bar is 5 µm.

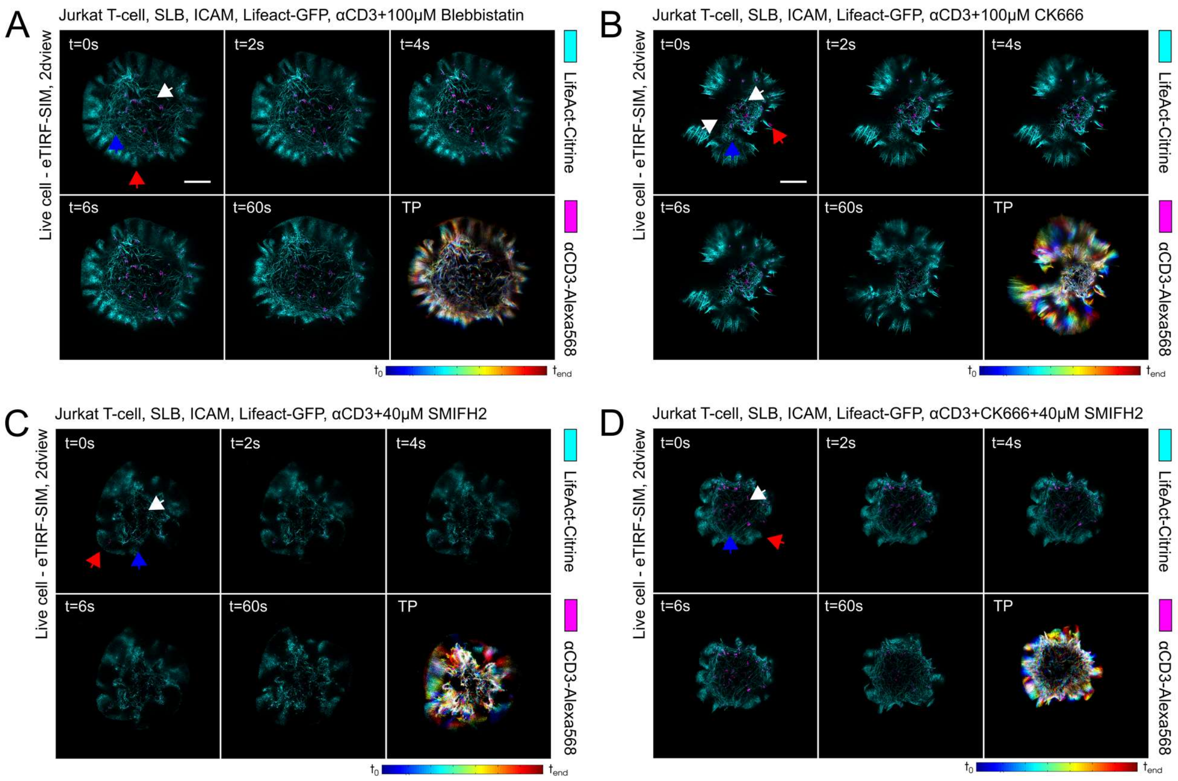

Fig. S3: Pharmacological treatments targeting actin cytoskeleton behaviour in immortalised CD4+ Jurkat T-cells. Representative TIRF-SIM images of the dynamics of (**A**) live Jurkat CD4+ T-cells expressing Lifeact-citrine and fluorescently labelled aCD3-Alexa568 at the basal membrane in response to 100 µM Blebbistatin, (**B**) 100 µM CK666, (**C**) 40 µM SMIFH2, and (**D**) the combination of 100 µM CK666 and 40 µM SMIFH2. The three characteristic F-actin networks including the lamellipodium (red arrow), lamellum (blue arrow), and ramified actin network (white arrow) are visible in the Jurkat CD4+ T-cells. Qualitative changes in the integrity of the lamellum region can be observed in response to Blebbistatin, the lamellipodium in response to CK666, and the synapse symmetry in response to SMIFH2 and the combination of 100 µM CK666 and 40 µM SMIFH2. Scale bar is 5 µm.

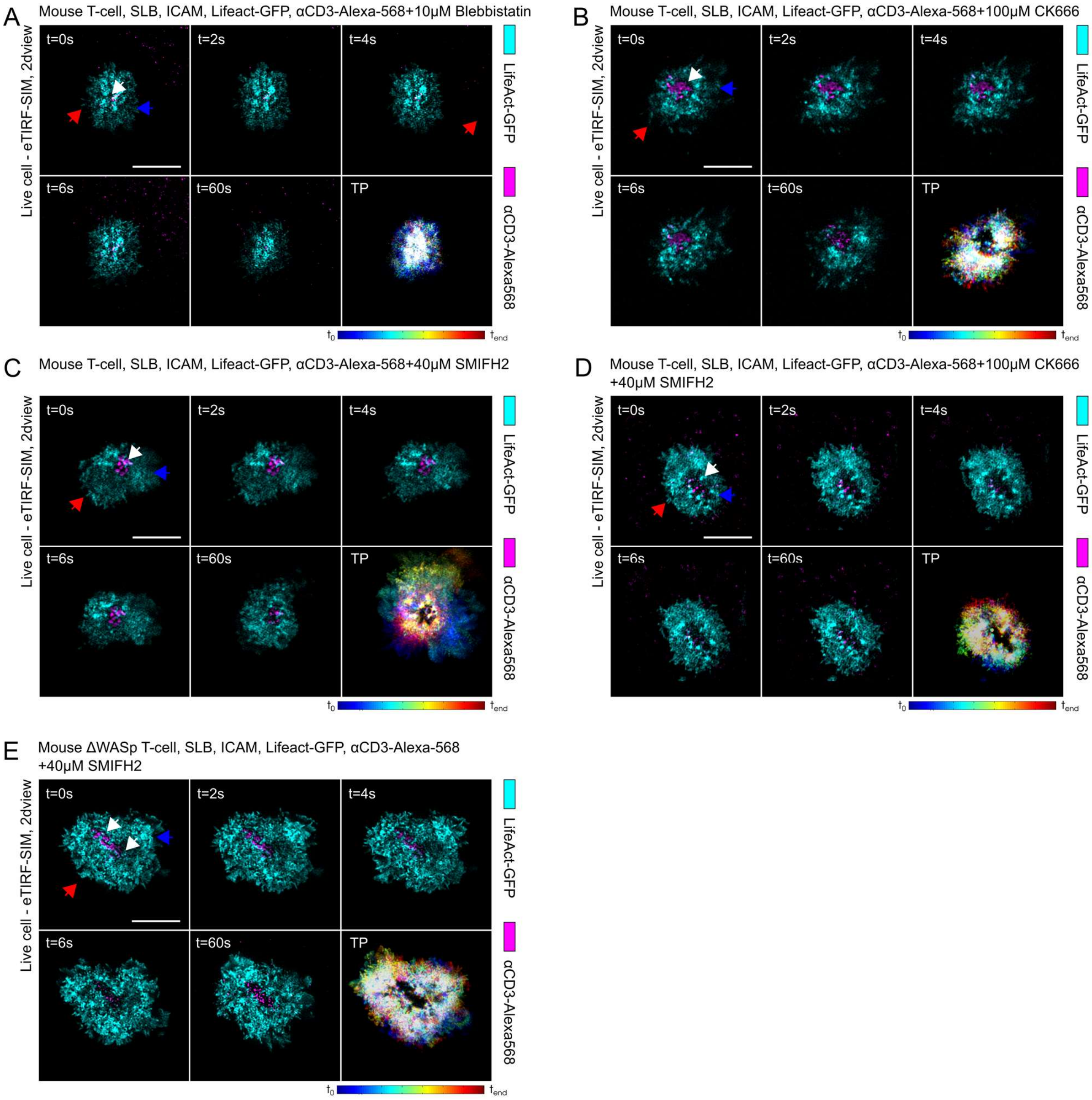

Fig. S4: Pharmacological treatments targeting actin cytoskeleton behaviour in primary CD4+ Mouse T-cells. Representative TIRF-SIM images of the dynamics of live Jurkat T-cells expressing Lifeact-citrine and fluorescently labelled aCD3-Alexa568 at the basal membrane in response to (**A**) 100 μM Blebbistatin, (**B**) 100 μM CK666, (**C**) 40 μM SMIFH2, and (**D**) the combination of 100 μM CK666 and 40 μM SMIFH2, and (**E**) *Δ*WASp + 40 μM SMIFH2. The three characteristic F-actin networks including the lamellipodium (red arrow), lamellum (blue arrow), and ramified actin network (white arrow) are visible in the Jurkat CD4+ T-cells. Qualitative changes in the integrity of the lamellum region can be observed in response to Blebbistatin, the lamellipodium in response to CK666, and the synapse symmetry in response to SMIFH2 and the combination of 100 μM CK666 and 40 μM SMIFH2 and *Δ*WASp + 40 μM SMIFH2. Scale bar is 5μm.

Journal of Cell Science • Supplementary information

Supplementary Movies

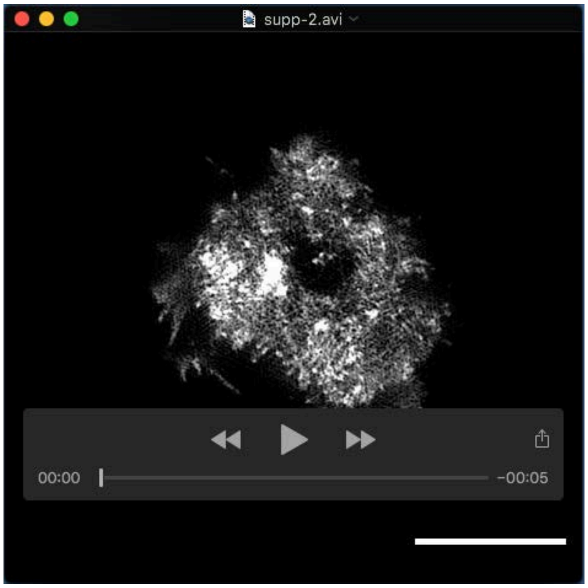

Movie 1: Representative TIRF-SIM images of live Mouse CD4+ T-cells expressing Lifeact-GFP. The frame-rate is 1 frame per second (fps). Scale bar is 5μm.

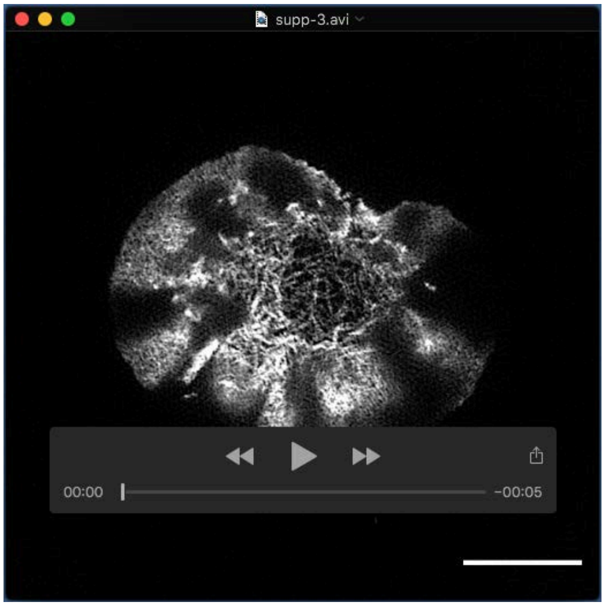

Movie 2: Representative TIRF-SIM images of live Jurkat CD4+ T-cells expressing Lifeact-citrine. The frame-rate is 1 fps. Scale bar is 5μm.

Journal of Cell Science • Supplementary information

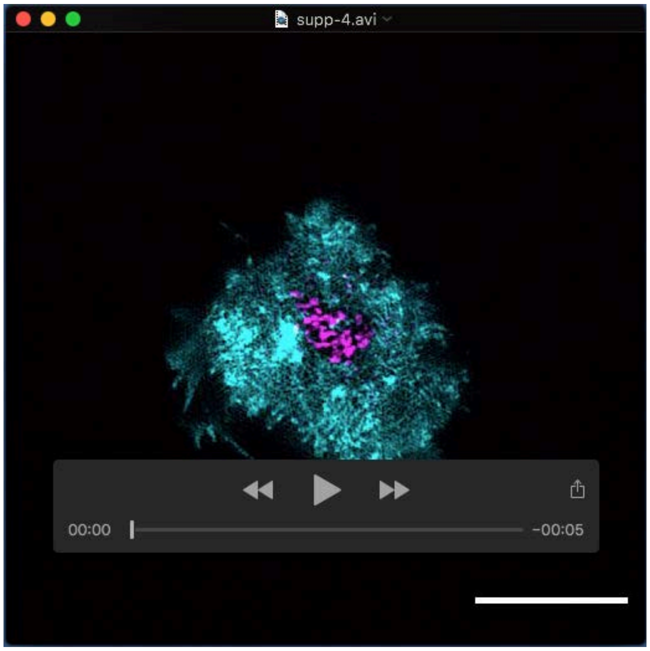

Movie 3: Representative TIRF-SIM images of live Mouse CD4+ T-cells expressing Lifeact-GFP. The T-cell receptor (TCR) is fluorescently labelled with aCD3-Alexa568. The frame-rate is 1 fps. Scale bar is 5μm.

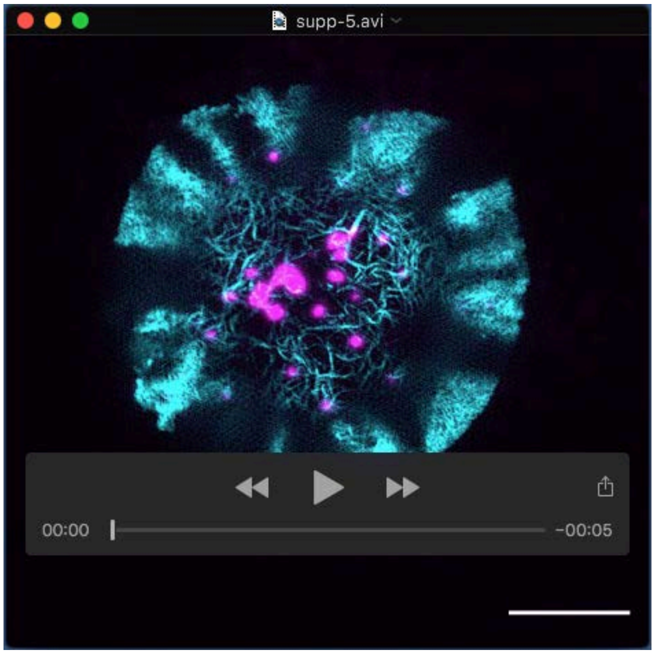

Movie 4: Representative TIRF-SIM images of live Jurkat CD4+ T-cells expressing Lifeact-citrine. The TCR is fluorescently labelled aCD3-Alexa568. The frame-rate is 1 fps. Scale bar is 5μm.

Journal of Cell Science • Supplementary information
